# Installation Season May Significantly Impact Time Required for Subterranean Termites to Find and Feed on In-Ground Baits

**DOI:** 10.3390/insects13050445

**Published:** 2022-05-07

**Authors:** Andrew M. Sutherland, Casey Hubble, Molly Barber

**Affiliations:** Division of Agriculture and Natural Resources, University of California, Davis, CA 95618, USA; cwhubble@ucanr.edu (C.H.); mabarber@ucdavis.edu (M.B.)

**Keywords:** rhinotermitidae, chitin synthesis inhibitors, bait interception time

## Abstract

**Simple Summary:**

Insecticide baits for use against subterranean termites have been shown to be highly effective, but the time required for termites to find and feed on baits may be a barrier to adoption in some areas. One explanation for this “time-to-attack” problem is that termite foraging near the soil surface may be limited during inhospitable periods. In California, characterized by a hot-summer Mediterranean climate, western subterranean termites have mostly been observed near the surface during the wet season, suggesting that baits installed in summer may sit uninvestigated for many months. To test this hypothesis, we established research plots in areas of known termite incidence, installing baits on four different dates over a one-year period and then recording termite activity every 60 days for two years. As expected, most foraging in these stations was observed in winter and spring. Time-to-attack for stations installed at the beginning of winter was significantly less than for stations installed at the beginning of summer (194 d vs. 296 d). These findings may help pest control operators in regions with pronounced dry periods to optimize their use of bait station systems by targeting specific installation seasons.

**Abstract:**

Rhinotermitid termites, serious pests of wooden structures throughout the world, are commonly controlled with chitin synthesis inhibitor bait systems. Seasonal termite foraging patterns in some regions may prolong bait interception time, however, significantly decreasing colony elimination speed. We hypothesized that installing baits immediately prior to the season of highest foraging activity will minimize interception time when baiting for *Reticulitermes* spp. in California, a region characterized by a hot-summer Mediterranean climate. To test this theory, we installed three different bait systems on four dates corresponding to the major seasons (spring, summer, autumn, winter) at five field locations known to harbor the target species. We then recorded initial termite discovery events every 60 days for two years, considering effects of installation season, bait system, site, and distance from previously observed termite incidence on bait interception time. Observed foraging activity in bait stations was highest during late winter and spring. Baits installed during winter exhibited interception times more than 100 days shorter than those of baits installed during summer. From these findings, we conclude that colony elimination speed and perceived CSI bait utility may be increased in Mediterranean climate regions when baits are installed immediately prior to the wet season.

## 1. Introduction

Subterranean termites (Blattodea: Rhinotermitidae), widely distributed in temperate and tropical regions worldwide, are the most significant of wood-destroying pests, causing more than USD 30 billion in damage and control expenses globally each year [1,2,3]. Commonly, subterranean termites have been deterred from attacking wooden structures by physical or chemical barriers placed under or around buildings [4,5]. Physical exclusion tactics, such as sand or other particle barriers, require maintenance and are prone to failure in some environments [4]. Chemical barriers, such as liquid termiticides applied to subsurface soil around structures, are commonly provided by pest control operators and may result in short-term control or repellency [4]. In some cases, however, liquid termiticides may repel termites from structures but fail to eliminate their colonies, meaning termites may return to attack treated structures when termiticide residues have degraded [6]. Baits for subterranean termites, consisting of cellulose matrices that contain slow-acting insecticides, have been considered valuable alternatives to liquid termiticides for decades [7]. Modern termite baits usually employ chitin synthesis inhibitors (CSIs), growth regulator chemicals that prevent insects from successfully forming new exoskeleton tissue, resulting in death during molting [1]. Subterranean termite workers may molt many times per year; in *Coptotermes formosanus* Shiraki, an estimated 1.7% of termite workers in a given colony molt each day [8]. Therefore, consumption of CSI baits by foraging workers (and subsequent spread throughout the colony via trophallaxis) has the theoretical capacity to eliminate the worker caste in as little as 60 d (100%/1.7% = 58.8). Soldiers, larvae, and reproductive castes reliant upon workers for nutrition eventually die of starvation [7]. Furthermore, new research [9] suggests that CSI consumption significantly reduces egg production by queens, egg size, and egg viability, accelerating colony elimination. Applications of CSI baits have been shown to eliminate entire colonies of rhinotermitid termites in both the laboratory [1,9] and the field [10,11], sometimes in periods as short as 60–90 d [12,13]. 

Time required for colony elimination is determined by three temporal factors: interception time, toxicant acquisition time, and lethal time [14]. The initial major determinant, *bait interception time*, or the time required for termites to find and begin feeding on bait, is highly variable and likely dependent upon foraging patterns [15]. Bait interception times reported by field researchers have varied from less than 60 d [11,16] to more than 400 d [17]. Once termites have been detected in bait stations, however, apparent colony elimination was usually reported in one year or less [10,18,19]. Long bait interception times may lead practitioners to conclude that colony elimination is too slow for CSI baits to be considered viable tactics for remedial pest control. In California, this conclusion has been shared with the authors by pest control operators as an explanation for the low industry adoption of baits in the region. California is characterized by a hot-summer Mediterranean climate [20], with almost all precipitation occurring during the cooler winter months. The primary target pest species in western North America, *Reticulitermes hesperus* Banks, is known to exhibit foraging patterns that likely correspond to seasonal differences in temperature and soil moisture [21]. In general, the observed pattern for *Reticulitermes* spp. in California is increased foraging activity during late winter and early spring months coupled with decreased foraging activity during autumn and early winter months [5,16]. This pattern may be pronounced in wildland areas as compared with irrigated urban areas [21]. Given that foraging activity drives initial termite discovery of potential new resources, we hypothesize that baits targeting *Reticulitermes* spp. in California that are installed at the beginning of winter will have reduced interception times as compared to those installed at the beginning of summer.

## 2. Materials and Methods

**Figure 1:**
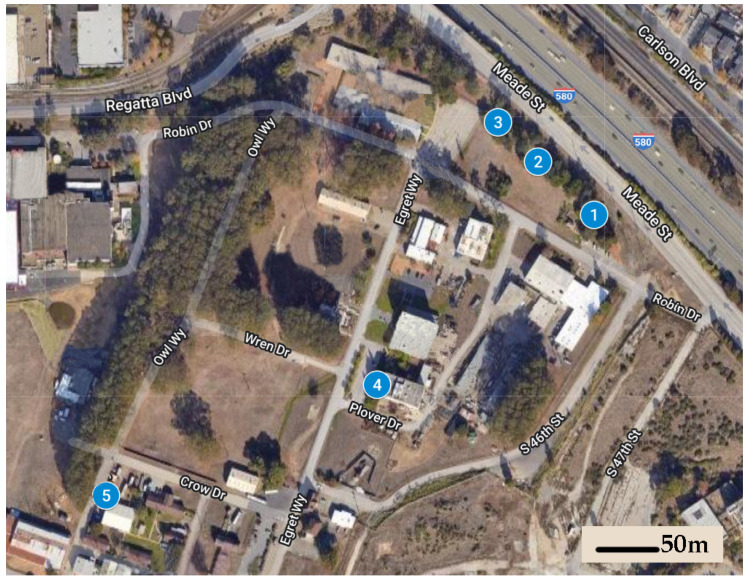
Five sites were established in 2019 at the University of California, Berkeley; Richmond Field Station; a sparsely-vegetated 40 ha property used for institutional and industrial purposes.

We established five field sites at the Richmond Field Station, a 40 ha University of California, Berkeley research station located 500 m from the San Francisco Bay, characterized by a mild Mediterranean climate with significant marine influences (temperature range: 6–24 °C, average precipitation = 63.4 cm). Average distance between sites was 160 m (range: 47–253 m) (Figure 1). Each of these sites was centered on a specific location where foraging subterranean termites, identified as *Reticulitermes* spp. (*R. hesperus* species complex, see [22]), were observed and collected during January 2019. 

**Figure 2:**
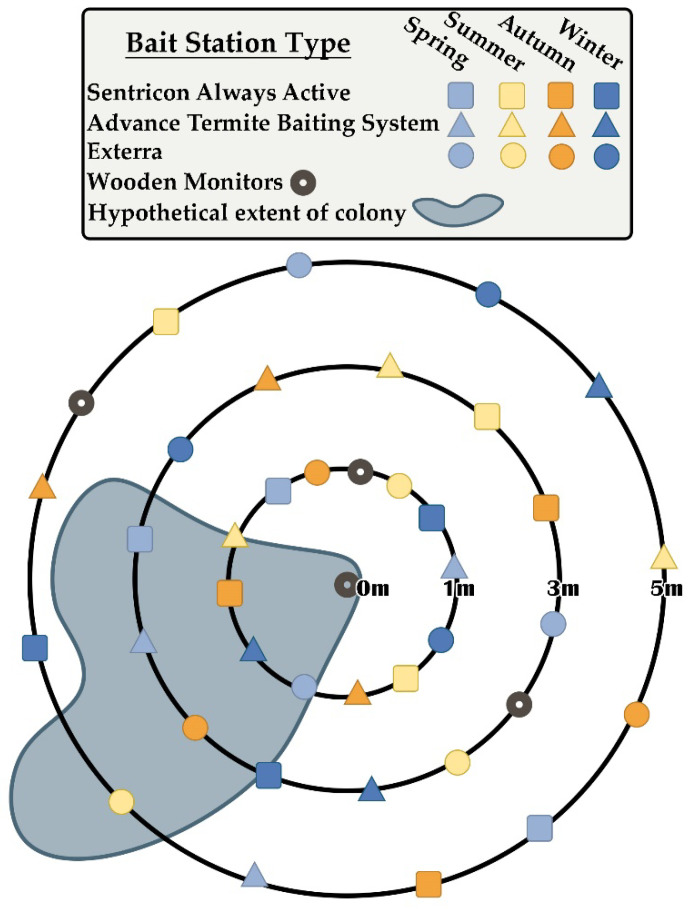
Representative example of study site arrays. Each site was centered on a specific location where subterranean termites had been observed and collected. Three bait station types were installed at three different distances from the site center during four different seasons during 2019 (see corresponding text for specific details and installation dates).

At each site, we installed stations from three different commercial CSI bait systems at the beginning of four different seasons and at three different distances from the central termite collection locations. Bait matrices within stations were provided by manufacturers and did not contain active ingredients. Bait systems represented included Advance Termite Baiting System (for use with Trelona Compressed Termite Bait: 0.5% novaluron, BASF SE. Florham Park, NJ, USA), Exterra (for use with Isopthor Termite Bait: 0.25% diflubenzuron, Ensystex, Inc. Fayetteville, NC, USA), and Sentricon Always Active (for use with Recruit HD Termite Bait: 0.5% noviflumuron, Corteva, Inc. Indianapolis, IN, USA). Installation dates roughly corresponded to seasonal events in the solar cycle (solstices and equinoxes): 25 March 2019 (spring); 24 June 2019 (summer); 23 September 2019 (autumn); and 16 December 2019 (winter). On these dates, one station from each bait system was installed along each of three concentric circles that were 1 m, 3 m, and 5 m from the centers of each site, for totals of 9 stations per site per season, 45 stations per season, and 180 bait stations overall (Figure 2). In addition, a monitoring station (Isopthor EZE; Ensystex, Inc.) containing wood (*Pinus* spp.) blocks was installed along each concentric circle and at the center of each site on 25 March 2019. All stations were installed so that circular access caps extended approximately 1 cm above the soil surface, with the remaining 20–25 cm underground and in continuous contact with the soil. Installation was accomplished using a ratcheting hand auger (18” Pro Series Ratcheting Cross Handle, 5/8” Thread, 3’ Extension Bar, 2 ½” Open-Face Auger, 2” Combination Edelman Auger; AMS, Inc. American Falls, ID, USA). Following each installation event, bait stations and monitoring stations were opened and examined for termite activity every 60 days [17] for two years. Bait interception time was considered to be the number of days between station installation and the first observation of feeding on bait matrices by the target species. In cases where termites were present during these inspections, approximately 30 individuals were collected in 95% ethanol for future analysis of molecular characters, especially those associated with colony fidelity [18]. Overall seasonal activity was described by considering the numbered Gregorian dates of initial termite discovery events during the two-year observation period and then continuously fitting these points using a nonparametric density estimation, executed using the *Analyze Distribution > Fit Smooth Curve* command within JMP Statistical Software [23,24]. Bivariate relationships between bait interception time and bait system, site, distance from site center, and bait system of nearest station were all considered using Wilcoxon signed-rank tests [25]. The bivariate relationship between bait interception time and distance from nearest station (a normally distributed continuous variable) was considered using linear regression. The effect of installation season on bait interception time was investigated by considering initial termite discovery events during the first year of observations only, since initial discovery events during the second year were invariably observed in stations that had already been in place for an entire seasonal activity cycle. The relationship between installation season and bait interception time was described using a general linear mixed model (residual maximum likelihood method), with installation season as the fixed effect and with bait system and site considered as random effects [26]. Least-squares means of bait interception times were compared amongst installation seasons using the Tukey honestly significant difference test. All analyses were conducted using JMP Statistical Software (JMP Pro 16, SAS Institute, Inc. Cary, NC, USA) [24].

## 3. Results

Feeding by *Reticulitermes* spp. termites was detected or observed within 43% (78 out of 180) of the bait stations and 45% (9 out of 20) of the monitoring stations installed. Foraging termites were encountered in 29 different stations, resulting in 22 voucher specimen collections.

### 3.1. Overall Seasonal Activity

**Figure 3:**
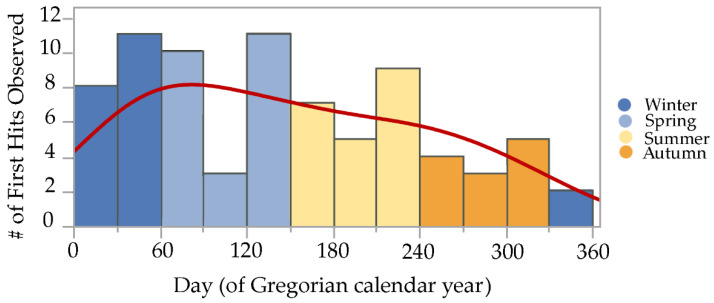
Initial discovery events or “first hits” by *Reticulitermes* spp. termites observed on cellulose bait matrices (without active ingredients) at five sites during 2019–2021 in the San Francisco Bay Area. Histogram colors demarcate the four Mediterranean climate seasons experienced in California: cool wet spring during days 61–150, hot dry summer during days 151–240, warm dry autumn during days 241–330, and cold wet winter during days 331–365 and 1–60. Red line over histogram shows output from a normal mixture density equation that continuously fits the data.

Initial termite discovery events, or *first hits*, were observed during all 12 months of both years, though there was a marked trend observed, with most first hits during late winter and spring, a gradual decline in first hits during summer and early autumn, and fewest first hits during late autumn and early winter. A normal mixture density equation using vectors for inspection month means, standard deviations, and probabilities generated a line that continuously fits these data, helping to illustrate the trends observed [23,24] (Figure 3). 

### 3.2. Observed Bait Interception Time

Two stations were found and fed upon within 60 days, and ten stations were found and fed upon within 120 days. Overall, however, the average bait interception time observed was greater than one year (367 ± 17.4 d, *n* = 78).

#### 3.2.1. Bivariate Relationships: Effects on Bait Interception Time

There was a significant bivariate relationship detected between site and bait interception time (Wilcoxon χ^2^ = 11.3, *p* = 0.02, df = 4), with overall times observed at Site 5 (292 ± 29.6 d, *n* = 22) significantly less than times observed at Site 4 (462 ± 46.3 d, *n* = 13); mean interception times observed at the other three sites were intermediate (all between 349 and 419 d) and statistically inseparable from the other sites. There were no detectable effects of bait system (Wilcoxon χ^2^ = 2.61, *p* = 0.27, df = 2; range: 327–383 d), distance from site center (Wilcoxon χ^2^ = 0.04, *p* = 0.98, df = 2; range: 356–377 d), bait system of nearest station (Wilcoxon χ^2^ = 0.30, *p* = 0.96, df = 2; range: 358–369 d), or distance from nearest station (R^2^ = 0.03, *n* = 78) on bait interception time. 

#### 3.2.2. Effect of Installation Season on Bait Interception Time

**Figure 4:**
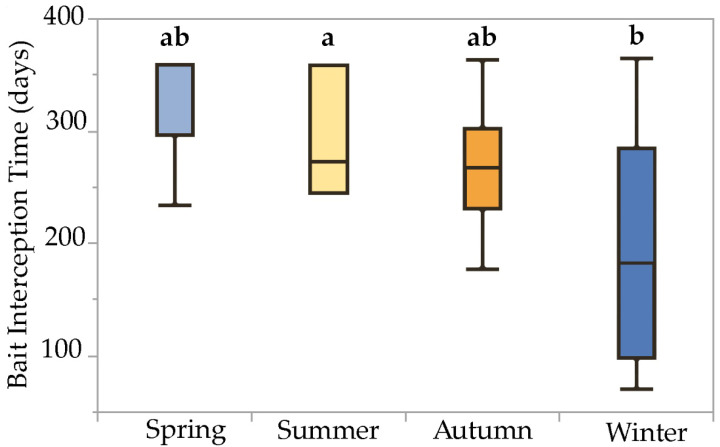
Boxplots illustrating statistical moments associated with bait interception time data collected over a one-year period from termite baits installed during four different seasons in the San Francisco Bay Area. Boxplots with the same letter code are not significantly different (Tukey HSD test on least-squares means, α = 0.05). Interception times for baits installed during winter were significantly less than those for baits installed during summer, while interception times for baits installed during spring and autumn were statistically intermediate between winter and summer.

The mixed model detected a significant fixed effect of installation season on bait interception time (F = 3.00, *p* = 0.04, df = 3) and attributed 9.38% and 0.53% of experimental variation to the random effects bait system and site, respectively. Comparison of least-squares means via Tukey’s HSD test revealed significant pairwise differences in bait interception time due to the installation season: bait stations installed on 16 December (winter) exhibited significantly lower interception times (194 ± 26.0 d, *n* = 9) than stations installed on 24 June (summer) (296 ± 24.7 d, *n* = 10). Interception times for stations installed on 25 March (spring) and 23 September (autumn) were statistically intermediate (282 ± 20.2, *n* = 15; and 268 ± 22.5, *n* = 12; respectively). This effect can be visualized using boxplots with means comparison letters, which show generally decreasing median interception times, as stations were installed progressively later in the calendar year, and a significant difference between stations installed in winter and summer (Figure 4).

## 4. Discussion

The main finding of this study was that the season of bait station installation significantly impacted observed bait interception times when targeting *Reticulitermes* spp. In California. It may be that seasonal differences in foraging near the soil surface created differential opportunities for termites to find and begin feeding on recently installed in-ground baits. Specifically, based on our observations, we hypothesize that foraging for new resources near the soil surface by *Reticulitermes* spp. May occur at the greatest frequency during late winter and early spring in California, reducing potential interception times for baits installed just prior to or during the early part of this seasonal period. Our observations are consistent with those previously conducted on *Reticulitermes hesperus* [21] and *Reticulitermes* spp. [16] in California. Soil moisture, soil temperature, and precipitation have been cited as environmental factors likely driving these seasonal activity patterns [21]. Significant differences in bait interception times by *Reticulitermes* spp. And *Coptotermes* spp. In different parts of the United States have also been attributed to environmental factors that vary amongst climatic regions [10]. Though not rigorously measured, air temperature and soil moisture varied widely from season to season during our trial, as expected. Summers were hot and dry, and rainfall occurred exclusively during cooler months. First significant rains were recorded during late November in 2019 and 2020 and during late October in 2021.

An alternative explanation for these results is that bait interception times decreased as cellulose resources (stations installed) within sites became more abundant. This was considered, but a clear linear trend based on this possibility was not detected. For instance, there were no differences in bait interception time between stations installed during spring (initial installation event) and winter (final installation event). Our experiment could be repeated, with different initial installation seasons, to confirm that the installation season rather than resource density was the factor driving interception time. 

There were significantly different bait interception times recorded among the five different study sites, which included differences in soil type, vegetation type, and irrigation regimes (see Figure 1). Mean interception time at Site 5, which was in unirrigated sandy soil near a wood building, was 292 d, while mean interception time at Site 4, which was in clay loam soil within an irrigated landscape bed dominated by coast redwood trees (*Sequoia sempervirens* (D. Don) Endl.), was 462 d. The other three sites, which were along a sporadically irrigated linear grove of pine (*Pinus* spp.) and oak (*Quercus* spp.) trees, exhibited intermediate interception times. Our experiment was not designed to determine the site factors that may influence bait interception time, but one hypothetical explanation for the differences observed is that there may have been much more cellulose debris in the landscape bed under the redwood trees than in the sandy and mostly unvegetated area adjacent to the old building, providing ample food for foraging termites and making the baits comparatively less important as resources. A potentially related observation was made with *Reticulitermes* spp. in Mississippi, where foraging termites were more likely to revisit monitoring stations in open grassland than they were in a forested habitat with presumed greater abundance of subterranean food resources [17]. The caveat to this finding was that stations in open grassland exhibited much higher initial interception times (up to 420 d) than stations in the forested habitat (as few as 90 d) [17]. The study referenced installed stations in areas where termite incidence had not been confirmed, while stations in our study were all installed in specific locations known to recently harbor termites.

One concern related to colony elimination speed is that some species of rhinotermitid termites may become repelled from bait stations by too-frequent inspections, which represent repeated disturbances. For instance, *Reticulitermes flavipes* (Kollar) workers were observed to take significantly more time to return to a food resource following a physical disturbance than *C. formosanus* workers [27]. Newer product labels, such as those associated with the bait systems included in this study, allow for inspection frequencies as low as once per year or once per six months, perhaps due to these findings and reports by practitioners. For this work, however, we opted to maximize our data set by utilizing a 60 d inspection frequency. This frequency has been compared to lower frequencies and was considered to have no significant effect on observed activity of *Reticulitermes* spp. workers in in-ground monitoring stations [17]. 

## 5. Conclusions

Overall, these findings may help pest control operators to optimize their use of bait station systems as subterranean termite control tactics by targeting specific installation seasons, especially in areas with pronounced dry periods, hot periods, or other periods considered to be inhospitable to foraging near the soil surface. These efforts may reduce bait interception times, leading to overall decreases in colony elimination time [14] and greater perceived efficacy within their client bases. All three of the bait systems represented in this study have been evaluated in the field and considered effective for remedial control of rhinotermitid pest species [10,19,28].

## Data Availability

The data presented in this study are available at the following Google Drive cloud storage link: https://docs.google.com/spreadsheets/d/1vUjxIuiNnXYsLB35C8Xhol7RB4Wd2DBQ/edit?usp=sharing&ouid=114980821805913012004&rtpof=true&sd=true, accessed on 31 March 2022.
